# In Vitro Engineering of Vascularized Tissue Surrogates

**DOI:** 10.1038/srep01316

**Published:** 2013-02-19

**Authors:** Katsuhisa Sakaguchi, Tatsuya Shimizu, Shigeto Horaguchi, Hidekazu Sekine, Masayuki Yamato, Mitsuo Umezu, Teruo Okano

**Affiliations:** 1 School of Creative Science and Engineering, TWIns, Waseda University, 2-2 Wakamatsu-cho, Shinjuku-ku, Tokyo 162-8480, Japan; 2 Institute of Advanced Biomedical Engineering and Science, TWIns, Tokyo Women's Medical University, 8-1 Kawada-cho, Shinjuku-ku, Tokyo 162-8666, Japan; 3 These authors contributed equally to this work.

## Abstract

*In vitro* scaling up of bioengineered tissues is known to be limited by diffusion issues, specifically a lack of vasculature. Here, we report a new strategy for preserving cell viability in three-dimensional tissues using cell sheet technology and a perfusion bioreactor having collagen-based microchannels. When triple-layer cardiac cell sheets are incubated within this bioreactor, endothelial cells in the cell sheets migrate to vascularize in the collagen gel, and finally connect with the microchannels. Medium readily flows into the cell sheets through the microchannels and the newly developed capillaries, while the cardiac construct shows simultaneous beating. When additional triple-layer cell sheets are repeatedly layered, new multi-layer construct spontaneously integrates and the resulting construct becomes a vascularized thick tissue. These results confirmed our method to fabricate *in vitro* vascularized tissue surrogates that overcomes engineered-tissue thickness limitations. The surrogates promise new therapies for damaged organs as well as new *in vitro* tissue models.

Tissue engineering methods have promised new cell-based therapies for defective organs through regeneration, and new tissue-analogue models for drug testing and biomedical and physiological research. However, facile procedures for preparing engineered 3-D tissues are made difficult by a lack of intrinsic vascularity and transport systems to nourish such engineered tissues: *in vitro* viable tissue-like systems often exhibit dimensions beyond practical perfusion limits, and have no functional blood vessels with flowing blood to supply nutrients and oxygen, and to remove waste products. Most approaches to engineer thick tissue are performed *in vivo* using the body of small animals as a living incubator to produce natural vascular networks^1^,^2^,^3^. This in-body reactor approach has many practical limitations for most envisioned applications.

Our laboratory has fabricated living cardiac cell sheets *in vivo*^4^,^5^. This entire process of fabricating living, dense cellular constructs, called “Cell Sheet Engineering”, has been demonstrated with several cell types^6^,^7^,^8^,^9^. Such layered cell sheets stratify tightly because of the preserved native extracellular matrix present on individual cell sheets, facilitating rapid establishment and maintenance of cell-cell communication among the layered cell sheets^10^,^11^. This cell sheet engineering technology has the potential to fabricate unique, functional cell-dense cell sheets for treating damaged and non-functional organs^12^ if problems including hypoxia, nutrient insufficiency, and waste accumulation^13^,^14^ are overcome. Therefore, cardiac cell sheets were transplanted into the subcutaneous tissue of nude rats, and these transplanted cell sheets were found to be preserved *in vivo* for extended time frames^5^. Key to such preservation and viability *in vivo* appears to be the adequate vascularization that allows host blood to perfuse the newly produced microvessels within the cell sheet. Rapid host perfusion supplies sufficient amounts of oxygen and nutrients to the implanted tissues. Therefore, to establish 3-D tissue engineering, nutrient and oxygen supplies which be perhaps supplied by biologically scavenging the host's vascular system, is essential. However, there is, as yet, no adequate bioreactor system to provide the necessary perfusion via vascularization in multi-layered cell sheets.

## Results

**Figure 1:**
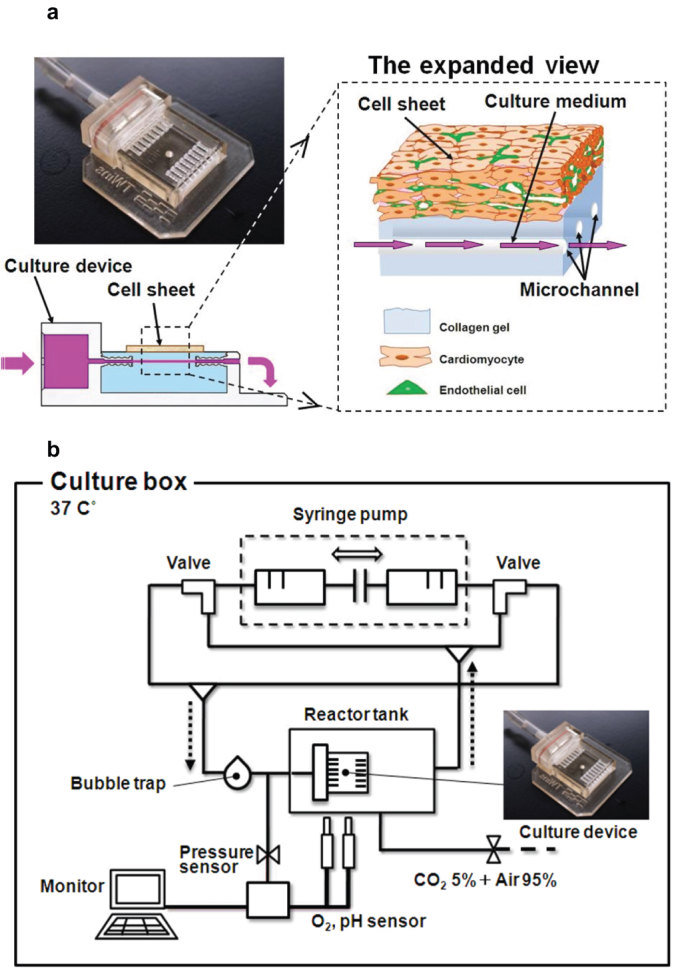
Culture device and perfusion culture system. (a) The collagen-gel base with microchannels imitates the conditions of a subcutaneous structure. In the right-hand expanded view, the blue area shows collagen-gel that can imitate a subcutaneous extracellular matrix, the pink arrows indicate the direction of culture medium flow imitate blood and tissue fluid. The culture medium can diffuse into the collagen gel and provide oxygen and nutrients to the cell sheet. The left picture shows a photograph of the culture device. (b) The circuit of culture system allows the culture medium to flow in the microchannels and monitors the pH and oxygen conditions of the culture medium as well as using partial pressure sensors. The culture medium was perfused into the culture device at 0.5 mL/min by a syringe pump. The oxygen and pH were monitored with an optic sensor to verify the conditions of the culture medium.

**Figure 2:**
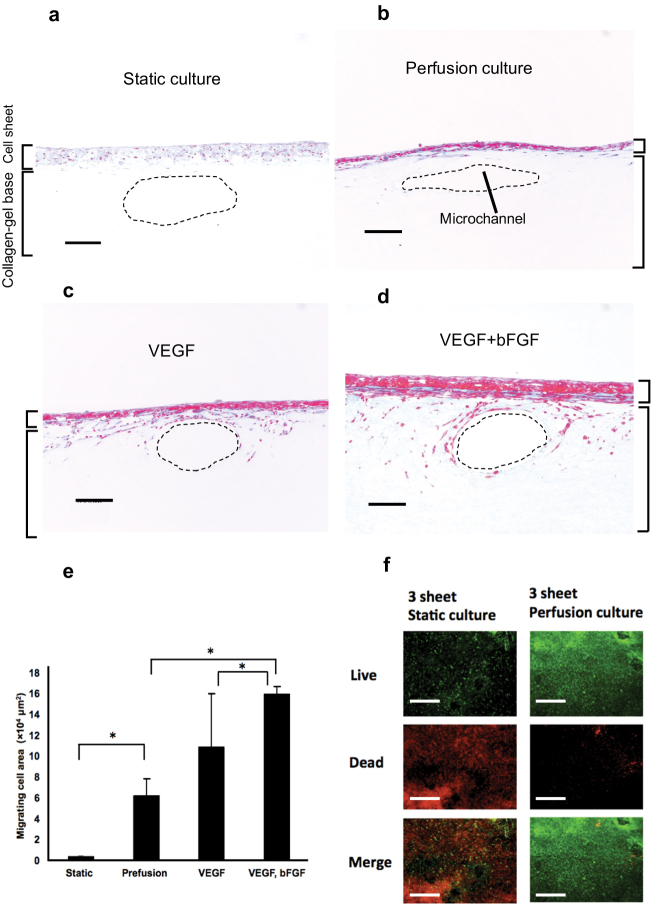
Cell migration from the cell sheet and tissue viability. The specimens were stained with AZAN. In the photographs (a)~(d), the upper layers are triple-layered cell sheets, and the lower part is collagen gel. The dotted-line circles are the microchannels. (a) Histological observation shows a triple-layered cell sheet on the collagen-gel cultured for 5 days without perfusion. After 5 days of static cultivation, the cell sheet had necrosed throughout. (b) A triple-layered cell sheet was cultured with perfusion at 0.5 mL/min for 5 days. The cell sheet construct was able to survive and several cells migrated into the collagen gel. (c) With the addition of vascular endothelial growth factor (VEGF), AZAN section showed that many cells had migrated and can be seen between the cell sheets and the microchannels. (d) VEGF and basic fibroblast growth factor (bFGF) stimulated triple-layered cell sheets on the collagen-gel. A large number of cells were found to migrate and create lumens in the collagen-gel base. (Scale bar, 100 μm) (e) Migrating cell areas were counted under the conditions in (a)~(d). The areas in perfusion culture systems were significantly larger than that in static culture. The areas in perfusion culture containing both VEGF and bFGF were significantly larger than those in perfusion cultures without two growth factors and only with VEGF. (*P < 0.05, n = 3) (f) Live/Dead assay was performed in the static culture and perfusion culture for the 3-layer cardiac cell sheet. Fluorescence micrograph shows cells stained by 5 μmol/L calcein-AM (green = live; top images) and 0.5 μmol/L ethidium homodimer-1 (red = dead; middle images). Bottom images are a merged version of the Live/Dead images. Most of the cells died in the static condition, consistent with the AZAN staining image. On the other hand, most of cells survived in the perfusion culture (Scale bar, 20 μm).

This study describes a newly designed approach to a bioreactor for engineering vascularized cardiac tissue based on the concept that layered cell sheets can culture successfully and reliably on an artificial culture having microchannels simulating *in vivo* subcutaneous transport and perfusion conditions. To realize this idea, a new perfusion bioreactor system for culturing 3-D cell-dense cardiac cell sheets on collagen-gel fabricated with imbedded microchannels able to mimic a subcutaneous vascular structure (Fig. 1a). After a triple-layered cultured cardiac cell sheet, that included endothelial cells, was placed over the collagen-base with microchannels, perfusion through the collagen-based microchannels was investigated, and effects of cytokines on vascularization were also studied. During incubation, a micro-syringe pump provided a constant flow of 0.5 mL/min to the bioreactor (Fig. 1b). After specific intervals of time under bioreactor perfusion, the cell sheet was removed and subjected to histological analysis. Specimens of these perfused cell sheets were stained by AZAN and compared to non-perfused cell sheet controls. AZAN sections of control cell sheets cultured without perfusion of culture medium demonstrated many faded color areas, indicating overall necrosis (Fig. 2a). By contrast, cell sheets with perfusion of medium showed both normal and healthy cell morphology within the cell sheets (Fig. 2b). In addition, triple-layered cell sheets were cultured with vascular endothelial growth factor (VEGF) in the culture medium. In that case, some migrating cells were observed (Fig. 2c). To better stimulate growth of vascular structures, cell sheets were also cultured in media that combined basic fibroblast growth factor (bFGF) and VEGF. Large numbers of cells migrated to create tubular structures with lumens within the collagen-gel base (Fig. 2d). Migrating cell areas in perfusion culture systems were significantly larger than that in static culture system. The areas in perfusion culture containing both VEGF and bFGF were significantly larger than those in perfusion culture systems without two growth factors and only with VEGF (Fig. 2e). To evaluate tissue viability, Live/Dead assay was performed in the static culture and in the perfusion culture with both VEGF and bFGF. Most of the cells died in the static condition in consistent with the AZAN staining image, whereas most of cells survived in the perfusion culture (Fig. 2f).

**Figure 3:**
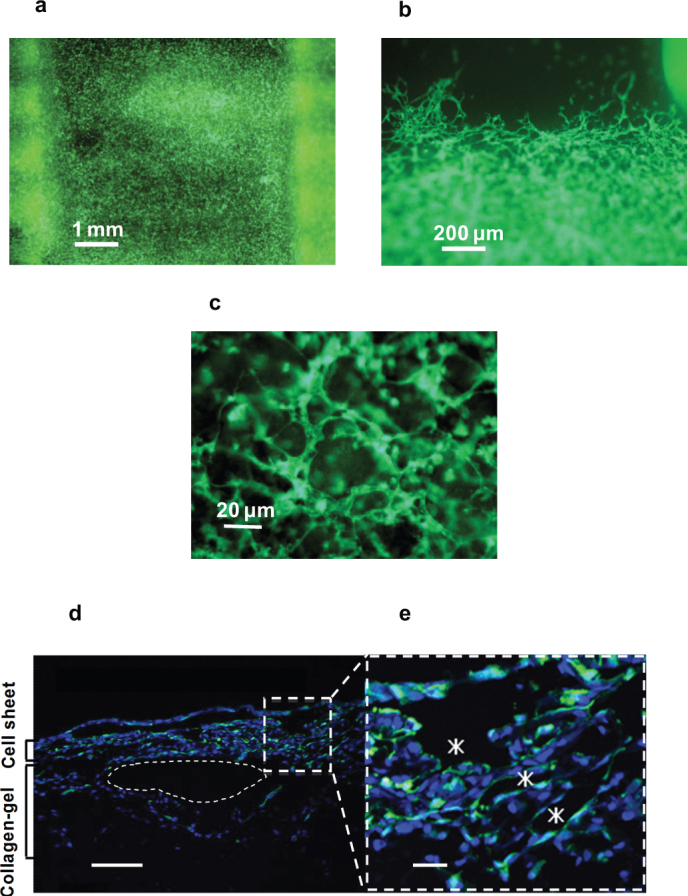
Geometry of endothelial cells of the cell sheet. (a)~(c) GFP-expressing endothelial cells were isolated from GPF neonatal rat heart and replaced with normal endothelial cells of the cardiac cell sheet by a magnetic cell sorter technique. These images are the top views of a 3-layer cardiac cell sheet after 5 days in perfusion culture under a microscope. GFP-expressing endothelial cells are seen as green. (a) Low magnification of the center of the construct. (b) Meddle magnification of edge in the construct. (c) High magnification of the center of the construct. (d) The specimen shows that GFP-positive cells exist at the lumen surface. Blue signals indicated nuclei. (Scale bar, 100 μm). (e) The right photograph shows the expanded view of the square area in (d) (Scale bar, 20 μm).

Next, green fluorescent protein (GFP)-expressing endothelial cells were replaced in culture-produced cardiac cell sheets to assist in identifying the appearance of endothelial cells. Co-cultured GFP-positive endothelial cells formed throughout the micro capillary network, as seen in the top views (Fig. 3a–c). In the cross-sectional views, GFP-positive endothelial cells cultured within the cardiac cell sheets were directly observed at luminal surfaces of newly formed microvessels in the constructs; similar to the luminal surfaces of microvessel vascular networks *in vivo* (Fig. 3d, e).

**Figure 4:**
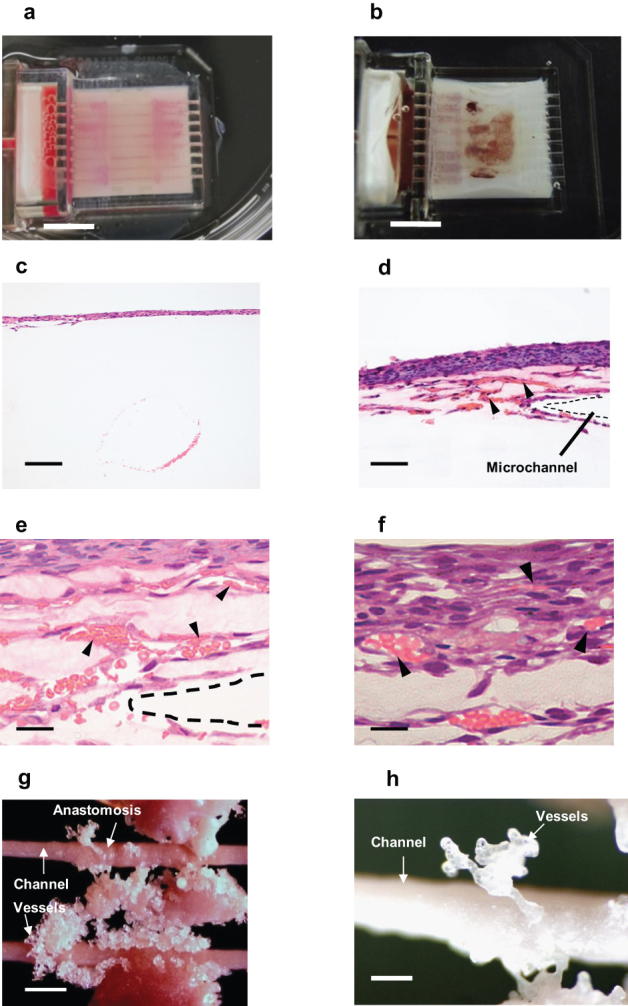
Red blood cells and resin perfusion into the endothelial lumen. (a) When the device has no endothelial cells, the flow line of red blood cells is clearly observed to follow on the microchannels (Scale bar 1 cm). (b) When endothelial cells are included, rat blood cells spread throughout the cell sheets like real subcutaneous vessels (Scale bar, 1 cm). (c) HE stained section shows no migrating cells in the construct without endothelial cells (Scale bar, 200 μm). (d) HE stained section of the construct containing endothelial cells shows a lot of migrating cells and a vascular formation. The red blood cells flowed into the newly created vascular network (Scale bar, 200 μm). (e) In the high magnification image, arrowheads indicate that red blood cells locate consistently to the lumens of the vascular networks between the microchannel and the cell sheet. The dotted line indicates the collagen-based microchannel (Scale bar, 50 μm). (f)Red blood cells locate in the cell sheet capillaries (Scale bar 20 μm). Arrow heads indicate the red blood cells. (g) The newly created vessel shape was cast by epoxy resin (Scale bar, 500 μm). (h) Expanded view of the resin cast of the newly created microvessels (Scale bar, 300 μm).

To verify the capillary-like fluid-carrying functions of the collagen microchannels, fresh rat red blood cells were perfused into the bioreactor immediately prior to the removal and fixation of the cell sheets from culture. In the bioreactor controls lacking endothelial cells, the microchannels are clearly observed as flow lines stained from the red blood cells (Fig. 4a). On the other hand cultured cell sheets containing endothelial cells show rat blood cells distributed across the cell sheets similar to actual subcutaneous vessels (Fig. 4b). After red blood perfusion, cultured cardiac cell sheets, with and without endothelial cells, were removed and analyzed histologically. Neither vascular structure nor red blood cell distribution was observed in the construct without endothelial cells (Fig. 4c). By contrast, red blood cells locate consistently to the lumens of vascular networks in the construct with endothelial cells, indicating that newly created vascular networks must spontaneously anastomose with the bioreactor's collagen-based microchannels to achieve connectivity (Fig. 4d–f). Moreover, in order to capture the shape of the newly created vascular networks, a cast was taken by perfusing an epoxy resin into the bioreactor. The cast of the vascular network showed that newly created vascular networks anastomosed with the bioreactor's collagen-based microchannels (Fig. 4g, h). This significantly demonstrates that: (1) the proposed and presumed newly created vascular networks observed within cultured cell sheets are indeed capable of reliable erythrocyte transport, just as capillaries can *in vivo*; (2) these cell sheet-based vascular networks effectively anastomose with the collagen-based microchannels in the bioreactor and exchange their fluid contents; and (3) fabrication of viable cardiac cell sheets with perfusable, functional vascular networks was successful. Significantly, this new capability will facilitate the production of thicker tissues.

**Figure 5:**
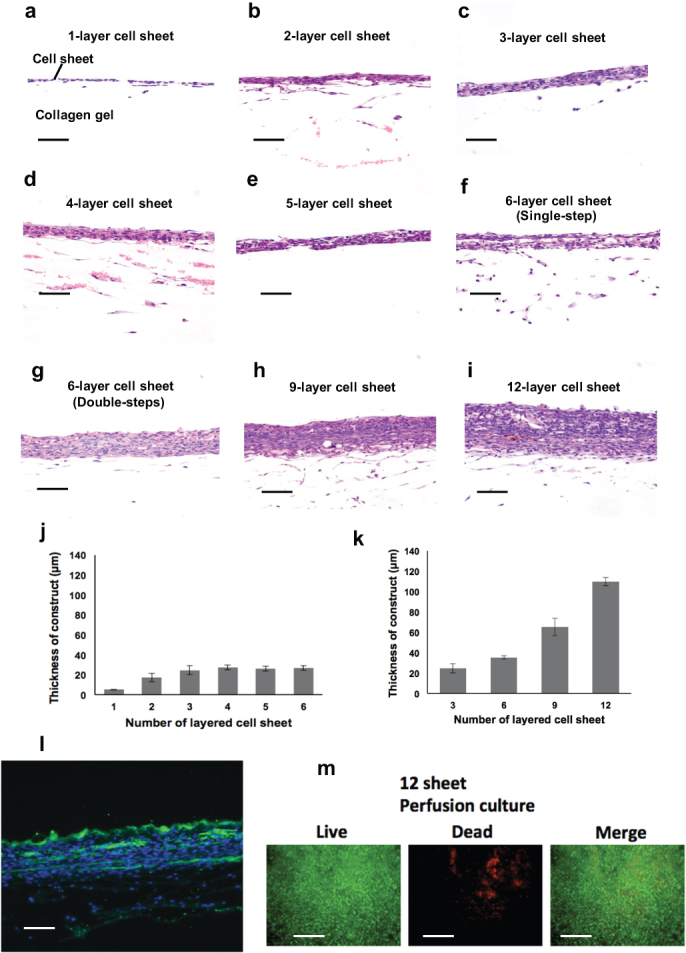
Fabrication of layered cardiac cell sheets on a perfusion bioreactor. In photographs (a)~(f), the upper layers are 1~6-layered cell sheets, and the lower parts are collagen gel. (a) The thickness single-layered cell sheet shows 5 ± 0.5 μm in the bioreactor after 5 days cultivation; (b) double-layered cell sheet is 17 ± 4 μm; (c) triple-layered cell sheet is 24 ± 4 μm; (d) quadruple-layered cell sheet is 27 ± 2 μm; (e) quintuple-layered cell sheet is 26 ± 2 μm; (f) sextuple-layered cell sheet is 27 ± 2 μm. (g) The thickness of the sextuple-layered cell sheet is 35 ± 2 μm. (h) The thickness of quintuple-layered cell sheet is 65 ± 8 μm. (i) Four triple-layered cell sheets layered up to four times at 5-day intervals produced a twelve layer sheet. The thickness of the twelve-layered cell sheet is 110 ± 4 μm. (a)~(i) (Scale bar, 50 μm). (j) Graph shows tissue thickness of the constructed grafts with a single-step layered procedure. The sheet thickness increased linearly up to triple-layered cell sheet and reached a plateau in quadruple to sextuple-layered cell sheets. (k) Graph shows that the tissue thickness of layered cell sheets increased up to a twelve-layered cell sheet with a multi-layered procedure. (l) Troponin T staining demonstrates the stratified cardiac muscle in a 6-layer cardiac cell sheet by the double-step procedure. Troponin T, Green; blue, nuclei (Scale bar, 20 μm). (m) Live/Dead assay was performed on the perfusion culture for the 12-layer cardiac cell sheet. Fluorescence micrographs show cells stained with calcein-AM (green-live; top images) and ethidium homodimer-1 (red = dead; middle images). Bottom images are merged from the Live/Dead images. A Live/Dead assay showed that most of the cells survived, but that focal cell death was observed (Scale bar, 20 μm).

Overcoming the intrinsic mass transport- and thickness-based limitations required for multi-layered cardiac cell sheet applications has been our focus, extending this vascular network proof-of-concept within the perfusion bioreactor to thicker sheets. Neonatal rat cardiomyocyte cell sheet constructs with varying numbers of cell layers (i.e., 1 to 6) were prepared and cultured individually. After 5-day culture in the perfusion bioreactor, each cell sheet construct was removed and fixed, and the thickness of each harvested construct was measured by microscopy (Fig. 5a-f). The total thickness of cell sheet constructs increases linearly up to 3 cell sheet layers, reaching a plateau for further cell sheet layering *in vitro* (i.e., 4–6 layers, see Fig. 5j). This was important to show the thickness limitations that hinder the fabrication of *in vitro* cell sheet-based tissue by layered cardiomyocyte sheets using a single-step sheet stacking and culture process.

To overcome this fabrication thickness limitation for layered cardiac cell sheets, a delayed interval layering culture process was performed with 3-layer cell sheets. First, a triple-layer cell sheet was cultured in the collagen-gel microchannel bioreactor for 5 days. The resulting sheet thickness was 24 ± 4 μm (n = 3). A second triple-layered cell sheet was then placed directly over the first 3-layer cell sheet in the bioreactor, yielding a 6-layer cell sheet, and this thicker cell sheet construct was further incubated in the bioreactor for 5 days (Fig. 5g). The 6-layer cell sheet was synchronously beating on the collagen-gel base (Supplementary Movie). Subsequently, a third 3-layer cell sheet was placed over the 6-layer cell sheet in the bioreactor, producing a 9-layer cell sheet. This thicker cell sheet construct was incubated again for another 5 days (Fig. 5h). This process was repeated again, yielding a 12-layer cell sheet, and incubated again for 5 days (Fig. 5i). This multilayer-stacking-culture experiment was repeated three additional times. During each layer stacking process, the average thicknesses of the 3-, 6-, 9-, and 12-layer cell sheet construct increased to 24 ± 4, 35 ± 2, 65 ± 8, and 110 ± 4 μm (n = 3), respectively (Fig. 5k). Histological analysis of anti-troponin T antibody staining revealed stratified cardiomyocytes in the 6-layer construct (Fig. 5l). HE stained sections of the multi-layer cell sheet constructs showed that the 12-layer cell sheet was well integrated without any observable necrotic tissue. A Live/Dead assay showed that most of the cells survived, but that significant cell death was focally observed within the 12-layer tissue (Fig. 5m). This multi-step layering procedure also provided vascularization within the cardiac tissue-like layers which exceeded 110 μm in thickness, well-beyond the diffusive transport limits for cells. During the initial 5-day incubation period for the first triple-layered cell sheet, endothelial cells from the cell sheet were observed to migrate into the collagen gel channels, forming vessels that eventually spontaneously anastomosed to the bioreactor microchannels enabling the requisite supply of media containing both oxygen and cell nutrients. Importantly, during further 5-day incubations after each new layer in the serial cell sheet stacking process (i.e., forming cell sheet constructs 6- to 12-layers thick), blood vessel precursor structures were directly observed throughout the constructs extending from the initial triple-layered cell sheet adjacent to the microchannels.

These key results for stimulating endogenous vascularization in an artificial cell culture system are unique, and provide critical new insights into strategies for producing clinically important tissue surrogates with new *in vitro* bioreactor designs, including: (1) *in vitro* engineered 3-D tissue surrogates with requisite biomechanical properties for eventually treating cardiac deficiencies, and (2) cardiac tissue-like models for applications in drug toxicity screening, biomechanics, transport physiology, improved tissue phantoms, and many other biomedical studies.

## Discussion

**Figure 6:**
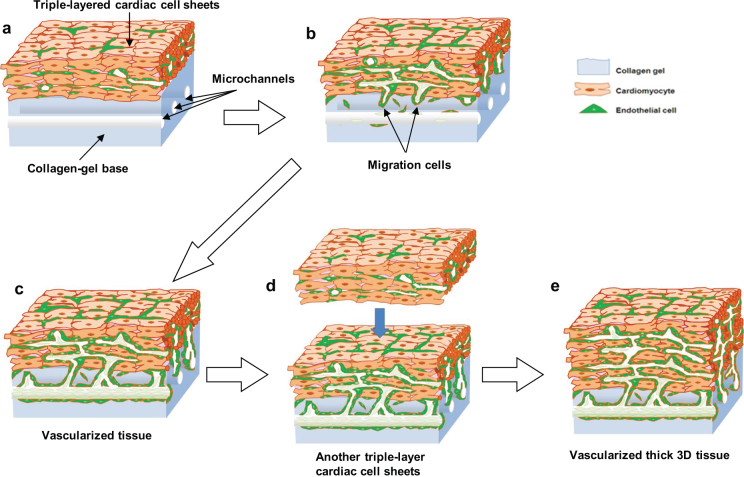
Fabrication process of vascularized cardiac tissue *in vitro*. The illustrations show the process of vascularization of multi-layer cardiac cell sheets on collagen-based microchannels. (a) A triple-layered cardiac cell sheet on a collagen-gel base with microchannels. (b) After 5 days cultivation with perfusion through the microchannels, endothelial cells migrated into the collagen gel and formed a lumen structure. (c) New lumen-like vascular network was able to connect to the collagen-based microchannels. Fresh medium could flow into the new vascular network and to the vessels in the triple-layer cardiac cell sheets. (d) After new microvessels formed connecting with the collagen-gel microchannels, another triple-layered cardiac cell sheet was placed on the existing cell sheet. (e) The newly-layered cell sheet spontaneously integrated with the existing cell sheet and was also rapidly infiltrated with budding vasculature extending from the previously vascularized cell sheets in the bioreactor. By repeating the sheet layering process, the subsequently added cell sheets were perfused with fresh medium through the newly-created vessels.

The process of functional microvessel formation in the constructs is illustrated in Fig. 6a–e. The illustrations show that endothelial cells from the cell sheets migrated to the bioreactor microchannels and consistently formed new vasculature-like structures in the collagen gel. This new vasculature eventually contributed substantially to cell transport, limited necrosis typically seen in thicker *in vitro* cultured cell masses, and provides new opportunities for improving ischemic limitations in regenerated tissue^15^,^16^. Evidence supports the utility of the bioreactor system to induce vasculature formation by applying both fluidic shear stresses^17^,^18^ and cytokine gradients within the collagen gel^19^,^20^. Essential to overcoming the recognized limitations of constructing 3-D tissues *in vitro*, triple-layer cell sheets were repeatedly layered over the pre-integrated cell sheets within the perfused reactor. Each subsequent cell sheet layer spontaneously integrated with the existing construct and was rapidly infiltrated with budding vasculature extending from the previously vascularized cell sheets in the bioreactor. As each new cell sheet layer was added it was perfused with fresh medium flowing through the newly created vessels. This unique microvessel cultivation and rapid recruitment into newly integrated cell sheets avoids necrotic complications and reliably nourishes the thicker cell sheets that would be unable to survive in diffusion-limited transport culture conditions.

We sequentially performed the multi-step procedure up to four times and successfully fabricated 12-layer constructs, however Live/Dead cell viability assays revealed focal cell death (Fig. 5m). When the procedures are repeated with the same cultivation conditions, the resulting vascular structure formation and media perfusion may be insufficient for whole tissue survival. Therefore, further biological and mechanical optimization (e.g. increasing flow rate in accordance with increasing tissue thickness) in the perfusion culture will be needed for making tissues thicker and more viable.

In summary, we have successfully fabricated thick, vascularized cardiac tissue-like surrogates with a new multi-step bioreactor and microchannel perfusion procedure *in vitro*. This approach spontaneously initiates then reliably builds an effective microvessel transport network between the growing cell sheets and the designed collagen-gel base. The *in vitro* bioreactor design improves on previously reported *in vivo* vascular bed formation for thick cell sheets^3^,^5^ by eliminating the tedious and impractical *in vivo* incubation steps. Importantly, the new method enables production of thick tissue constructs with pre-integrated and vessel-populated architectures that should provide new opportunities to contribute to more effective organ engineering, including highly perfused, high metabolic capacity tissues in the heart, liver, and kidney. This strategy is presented to contribute several new design and creation concepts, as well as practical production insight for future *in vitro* vascularized tissue surrogates, promising significant advances to both preclinical and clinical applications of tissue engineering.

## Methods

All animal experiments were performed according to the “Guidelines of Tokyo Women's Medical University on Animal Use”. (The institutional approved ethics protocol # 12–10).

### Preparation of neonatal rat cardiac cell sheets

Neonatal rat cardiomyocytes were isolated from ventricles of 1-day-old rats (CLEA, Tokyo, Japan) using previously reported procedures^21^. The resulting cell suspensions were seeded at a density of 3.6 × 10^6^ cells/dish on a temperature-responsive cell culture dish (UpCell^TM^ dish, I.D. 35 mm, Type-E, CellSeed, Tokyo, Japan). Seeded dishes were incubated for 4 days. Confluent cells were harvested as a uniform single, intact and viable cell sheet by incubating the culture dishes in a CO_2_ incubator set at 20°C for 1 hour^22^,^23^.

### Collagen gel support base with microchannels

Stainless steel wires (diameter 300 μm) were inserted into holes (I.D. 400 μm) in a culture device made by a rapid prototyping system (EDEN) (Objet Geometries, Billerica, MA, USA) (Fig. 1a). Neutralized collagen solution (0.5%, made from 10% decuple-concentrated cardiac cell culture medium described above), 10% balanced solution containing 10 mmol/L Hepes (Sigma, St. Louis, USA), 15 mmol/L NaHCO_3_, and 0.5% collagen Type-1 (IAC-05) (KOKEN, Tokyo, Japan) was poured into the culture device with aligned stainless wires held in parallel. To obtain a hard collagen gel, the collagen solution was incubated for 30 min at 37°C. The steel wires were extracted from the mold after collagen gelation, yielding 300 μm-diameter channels in the collagen gel. Simultaneously with the collagen gelation, the first triple-layer cell sheet was placed on the collagen gel base containing the microchannels. The distance from the gel-formed microchannels to the overlaying cell sheets was approximately 500 μm.

### Bioreactor assembly and perfusion culture condition

To stabilize the collagen-gel base, the collagen-gel base with the 3-layer cell sheet was cultured aseptically at 37°C for 1 hour. Next, the collagen-gel base was connected to a micro-syringe pump (KDS270) (KD Scientific, Holliston, USA). All bioreactor devices were placed in a culture box (As one, Tokyo, Japan) and maintained at 37°C by a fan heater (Sankei, Tokyo, Japan) then supplied with a mixed gas to maintain pH 7.4 (5% CO_2_, 95% air). The perfusion rate of the culture medium was 0.5 mL/min (Fig. 1b). The perfusion culture medium was comprised of 6% fetal bovine serum, 40% medium 199, 1% penicillin-streptomycin solution, and 54% balanced salt solution: containing 166 mmol/L NaCl, 1.0 mmol/L NaH_2_PO_4_, 0.8 mmol/L MgSO_4_, 26.2 mmol/L NaHCO_3_, 0.9 mmol/L CaCl_2_, 5 mmol/L glucose, 50 ng/mL bFGF, and 50 ng/mL VEGF.

### Histological analysis

After removal from the culture devices, multi-layer cell sheet constructs were fixed with 4% paraformaldehyde (Wako Pure Chemicals, Osaka, Japan) and routinely processed into 10-μm-tick paraffin-embedded sections. Hematoxylin and eosin, and Azan stained sections were prepared by conventional methods and examined by optical microscope.

To confirm perfusable blood vessels, a 1:10 diluted rat blood was perfused. For detecting GFP expressing endothelial cells, the constructs were frozen and sectioned. To detect cardiomyocytes, cross-sections were immunolabeled with a 1/100 dilution of anti-troponin T antibody (Thermo Scientific, CA, USA) for 2 hours at RT, and secondary stained for 2 hours at RT with Alexa Fluor 488-conjugated anti-mouse IgG anti- body for green fluorescence.

### Analysis of migrating cells

The images of the AZAN-stained sections of the constructs were taken in situ by a microscope (ECLIPSE E800, Nikon, Tokyo, Japan). The area containing cells that migrated from the cell sheet was measured with ImageJ software. All data are expressed as mean ±SD. An unpaired Student's t-test was performed to compare the two groups. One-way ANOVA was used for multiple group comparisons. If the F-distribution was significant, a Fisher's LSD test was used to specify differences between groups. A p-value of less than 0.05 was considered significant.

### Live/Dead assay for process viability

A layered cell sheet was used to characterize cell viability after the fabrication process. After cultivation in the bioreactor, the constructs were submerged in a Live/Dead staining solution (5 μmol/L calcein-AM-green and 0.5 μmol/L ethidium homodimer-1, Invitrogen, CA, USA) in a pH-adjusted buffer. The submerged constructs were allowed to incubate for 30 minutes at 37°C and 5% CO_2_. Fluorescence images were acquired using a microscope (OV-110, Olympus, Tokyo, Japan).

### Separation of endothelial cells from primary isolated cardiac cells

To observe the behavior of endothelial cells in the myocardial cell sheets, the endothelial cells were first removed and replaced with GFP-expressing endothelial cells by magnetic cell sorting (MACS). For separating endothelial cells from normal rats or GFP-expressing rats (SLC, Hamamatsu, Japan), primary myocardial cell suspensions were incubated with mouse monoclonal anti-rat CD31 antibody. After rinsing with running buffer (PBS containing 5% BSA and 2 mM EDTA), cells were incubated with anti-mouse IgG-conjugated microbeads (Miltenyi Biotec GmbH, Bergisch Gladbach, Germany) and then washed again with running buffer. The labeled cells were applied to a LS column in the magnetic fields of a MiniMACS system (Miltenyi Biotec GmbH, Bergisch Gladbach, Germany).The column was then washed with running buffer and removed from the magnetic fields, and the trapped cells in the column were flushed out with running buffer. Endothelial cell-depleted cardiomyocyte suspensions were used to create cell sheets without endothelial cells. To fabricate myocardial cell sheets containing GFP-positive endothelial cells, GFP-negative cardiac cells (without endothelial cells), and purified GFP-positive endothelial cells were mixed in a ratio of 8:1.

### Resin perfusion for observation of lumen shape

To create a cast of the shape of the newly created vascular networks, an epoxy resin (MERCOX II, VT, USA) was perfused into the microchannels in the collagen-gel base. This resin is a modified acrylic casting material consisting of two components, a resin and a catalyst. After the liquid resin was mixed with a catalyst (20:1), it was perfused into the microchannels for 5 minutes, then the constructs were treated using 5% NaOH to remove the collagen-gel and the cell sheets.

## Author Contributions

K. Sakaguchi designed and conducted experiments, analyzed data and wrote the paper. T. Shimizu designed and conducted experiments, analyzed data and wrote the paper, and supervised this project. S. Horaguchi and H. Sekine designed and conducted experiments. M. Yamato, M. Umezu, and T. Okano supervised the project.

## Supplementary Material

Supplementary Information Supplementary movie
